# Enhancing patient accessibility of primary care: the redesign of Italian territorial medicine

**DOI:** 10.1007/s10729-025-09721-x

**Published:** 2025-10-04

**Authors:** Antonio Diglio, Chiara Morlotti, Giuseppe Bruno, Mattia Cattaneo, Stefano Paleari, Carmela Piccolo

**Affiliations:** 1 Department of Industrial Engineering, University of Naples Federico II, Piazzale Tecchio 80, 80125 Napoli (NA), Italy; 2 Department of Management, Information and Production Engineering, University of Bergamo, Via Pasubio 7B, Dalmine, 24044 (BG) Italy

**Keywords:** Healthcare services, Healthcare management, Community healthcare centers, Primary care, General practitioners

## Abstract

Ensuring widespread accessibility of healthcare services is a crucial policy objective. Accordingly, the Italian National Recovery and Resilience Plan (NRRP) has prioritized territorial medicine, channeling post-pandemic investments toward the restructuring of primary care services. A notable change is the establishment of Community Healthcare Centers (CHCs). This paper investigates how CHCs contribute to the accessibility of healthcare in urban and rural areas. By leveraging a comprehensive dataset of general practitioners’ availability and estimating future demand-and-supply scenarios, we examine the impact of CHCs under two different capacity allocation strategies. *Strategy 1—Capacity expansion*—involves allocating additional service hours of general practitioners to CHCs in order to maximize accessibility. *Strategy 2—Capacity redistribution*—accounts for the persistent shortage of healthcare professionals faced by Italy in the recent years by reallocating a portion of general practitioners’ current services from their existing workplace locations to CHCs. Our results indicate that CHCs have the potential to maintain current accessibility levels and also enhance them in the years to come. Moreover, we demonstrate that simply redistributing the current capacity can improve future accessibility. Finally, we show that a mix of the capacity expansion and redistribution strategies (*Strategy 3*) can maximize accessibility in the future, limiting the need for new professional staff.

## Highlights

- Levers of future variations in accessibility of primary care are investigated.
- Community Healthcare Centers contribute to enhancing future accessibility of primary care
- Capacity expansion and redistribution are analyzed as alternative strategies.
- Improvement in future accessibility can be achieved even by redistributing capacity.

## Introduction

Globally, healthcare systems have consistently undergone pivotal reforms to improve the efficient delivery of care services, demonstrating adaptability to evolving economic and societal conditions. A significant reassessment of healthcare systems followed the COVID-19 pandemic, shedding light on their structural weaknesses worldwide, including the frailties of local health systems and the failure of the hospital-centric organizational model. To cope with this crisis and revive member states’ economies, the European Commission supported significant investments under the Next Generation EU framework to make health systems more efficient and resilient. Italy was at the forefront of implementing a massive reform process in this context. The *Italian National Recovery and Resilience Plan* (*NRRP*), in fact, allocates substantial funds to healthcare (Mission 6), identifying the reinforcement of primary care as a strategic development goal (Component 1 of Mission 6 ”*Proximity networks, structures and telemedicine for community healthcare*” - see [26]). In May 2022, the Italian government issued the Ministerial Decree nr. 77/2022 (”*Models and Standards for the Development of Community Care in the National Health Service*” - see [27]) to strengthen the provision of primary care services across all regions and overcome disparities that emerged in the last decades as a result of the ongoing political decentralization of the Italian National Healthcare Service - NHS.

This new reform proposes a standard organization model for primary care by defining the type of facilities to include in the proximity networks and the services they should offer [35]. Specifically, the envisaged model incorporates new key institutions, commonly referred to as *Case di Comunità* (*Community Healthcare Centers - CHCs*). These are socio-healthcare facilities designed to provide prevention services, continuity of care, and 24/7 availability in a more accessible and equitable manner, through the engagement and collaboration of various healthcare professionals, such as general practitioners (GPs), nurses, and specialists. From a regulatory perspective, CHCs are expected to yield guaranteed improvement in the accessibility of primary care services. In fact, the reform foresees the presence of a CHC per 50,000 inhabitants and requires each CHC to provide continuous medical assistance (24 hours a day).

Although promising, this reform must address two main challenges. The most significant difficulty is the staffing of new facilities and services given the shortage of healthcare workers [11]. Notably, in the past five years, Italy has registered a 7.5% decrease in the number of GPs for every 10,000 inhabitants [25]. Under these conditions, the feasibility of the new primary care model is at risk, and regions could encounter severe hardships in implementing the plan. The second challenge lies in ensuring good accessibility of primary care services. This new model risks to heighten the *concentration* of GPs (from dispersed ambulatories to CHCs) due to the above-mentioned trend. Hence, informed recommendations and studies on possible implementation strategies would be beneficial for local authorities.

Given this backdrop, this paper aims to investigate how the activation of CHCs influences the accessibility of primary care services in the Italian NRRP context. Focusing on Piedmont, the second largest Italian region in terms of geographical extension, we first define our baseline estimates by assessing the actual level of accessibility in urbanized and rural areas both (i) under current conditions, and (ii) considering the expected changes to major future trends, namely demographic decline, population aging, and contraction in healthcare resources. Then, we develop different hypotheses for the staffing of new healthcare facilities (i.e., CHCs). First, we consider staffing the CHCs by ensuring the availability of more GPs (*Strategy 1 - Capacity expansion*). Next, we assume mandating the existing GPs to deliver their services within CHCs (*Strategy 2 - Capacity redistribution*). Finally, we also devise a hybrid strategy (*Strategy 3*), where both elements of capacity expansion and redistribution are simultaneously considered. The strategies are evaluated in terms of their impacts on patients’ accessibility.

Apart from a practical standpoint, the problem at hand is relevant from an academic perspective. In fact, unlike research on primary-care settings (see, e.g., [20, 49]), the literature on CHCs’ accessibility is still scant and developing gradually. It is worth noting that CHCs are relatively recent initiatives implemented across NHSs worldwide [5, 18, 21, 46]. Further, although broadly accepted, the term ”Community Healthcare Center” does not have a universal definition, and the naming of these facilities varies from country to country. However, the overarching goal remains consistent: to enhance access to primary care services, especially in underserved areas. As a result, past (and ongoing) academic efforts on the topic mainly target policy-related aspects, delving into the reforms that led to their implementation and their implications for equity of access (i.e., their actual use — see, e.g., [42] and the references therein). Only a few studies explicitly deal with the geographic (or spatial) accessibility of CHCs, that is, an analysis of their territorial distribution and their reachability for patients. In these studies, the focus is mainly on analyzing the impact of CHCs on equitable accessibility conditions in rural areas [2, 9, 15, 36, 50, 51], and among more vulnerable populations, such as the elderly [12, 50, 52], racial minorities [44, 50], and low-income residents [17]. Other lines of research include the (positive) influence of CHCs’ accessibility on the use of primary care services or health outcomes [29, 31], a comparative assessment of patients’ accessibility in pre vs. post-health-reform scenarios [40], and the analysis of how accessibility varies depending on modes and times of transportation [38]. Some studies have also employed a decision-making perspective in their analysis, adopting basic facility location models for the optimal siting of CHCs to maximize accessibility [10, 47].

To the best of our knowledge, the existing literature focuses on already implemented health reforms and is mainly limited to descriptive-type analysis. Little (or no) attention is paid to the implications of optimal staffing policies at CHCs on patients’ accessibility and how these policies can be shaped depending on the current and projected primary care landscape. Given this context, this paper contributes to the literature in three ways: (i) by performing an extensive accessibility analysis of GPs’ primary care networks, assessing both current and future variations, as well as local disparities between urban and rural areas; (ii) devising and modeling alternative staffing strategies at CHCs based on new GPs’ allocation and re-allocation decisions; (iii) and assessing, by means of a fine-grained empirical study and a computational experiment, the role of CHCs in reinforcing patients’ accessibility in one of the Italian regional health systems.

The remainder of this paper is structured as follows. Section 2 details the methodology employed to measure accessibility and provides an overview of the case study investigated. Section 3 presents the first accessibility results derived from our analysis, providing current and future estimates. Section 4 investigates the role of CHCs, providing evidence of how the different strategies may impact accessibility. Finally, Section 5 concludes the paper with a summary of the work, a discussion of the main results and an outline of future research directions.

## Measure of accessibility

Recognizing equity of accessibility of healthcare services as a key policy goal for governments and national healthcare institutions, numerous studies have examined the potential discrepancy between the supply and demand of such services. Different methodological approaches have been proposed. Some of the common metrics used in the previous literature are the distance to the nearest healthcare facility (e.g., [1]) and the minimum travel time required for a patient to reach it (e.g., [13]). Although these two simple measures find their natural applications in hospital network design and emergency services, estimating the accessibility of non-urgent healthcare requires accounting for both service capacity and the volume of patients seeking care [28, 48]. Gravity models that jointly account for demand, supply, and distance were the starting point for such estimates [22], and over the past few decades, scholars have proposed various advancements to these approaches [32, 33, 45]. An effective and robust improvement to gravity models, widely used to measure accessibility of primary healthcare services, is the two–step floating catchment area method (2SFCA—see [37] for an extensive review on the topic). This method consists of two main estimations. Let *I* be the set of patient locations (indexed by *i*) expressing the potential demand for primary health services ($u_i$) and *J* the set of current healthcare facilities with capacity $w_j$. With $d_{ij}$ being the distance from patient location *i* and healthcare facility *j*, the first step in a 2SFCA estimates the supply-to-demand ratio at each healthcare facility ($R_j$). This ratio is computed as the capacity of facility *j* divided by the population at location *i* ($u_i$) living within a certain threshold distance $d^*$:

1 $$\begin{aligned} {R_j= \frac{w_j}{ \sum _{i \in I:{d_{ij}\le d^*}} u_i}}. \end{aligned}$$

The second step takes a population perspective and for each location *i*, it computes the sum of all the capacity-to-population ratios of the healthcare facilities located within the same distance threshold $d^*$. This value represents the spatial accessibility ($A_i$) of each location, and it is equal to:

2 $$\begin{aligned} A_i={ \sum _{j \in J:{d_{ij}\le d^*}} R_j}. \end{aligned}$$

For easier reference, all the notation utilized in the paper is listed in Appendix A—Table 7.

**Fig. 1:**
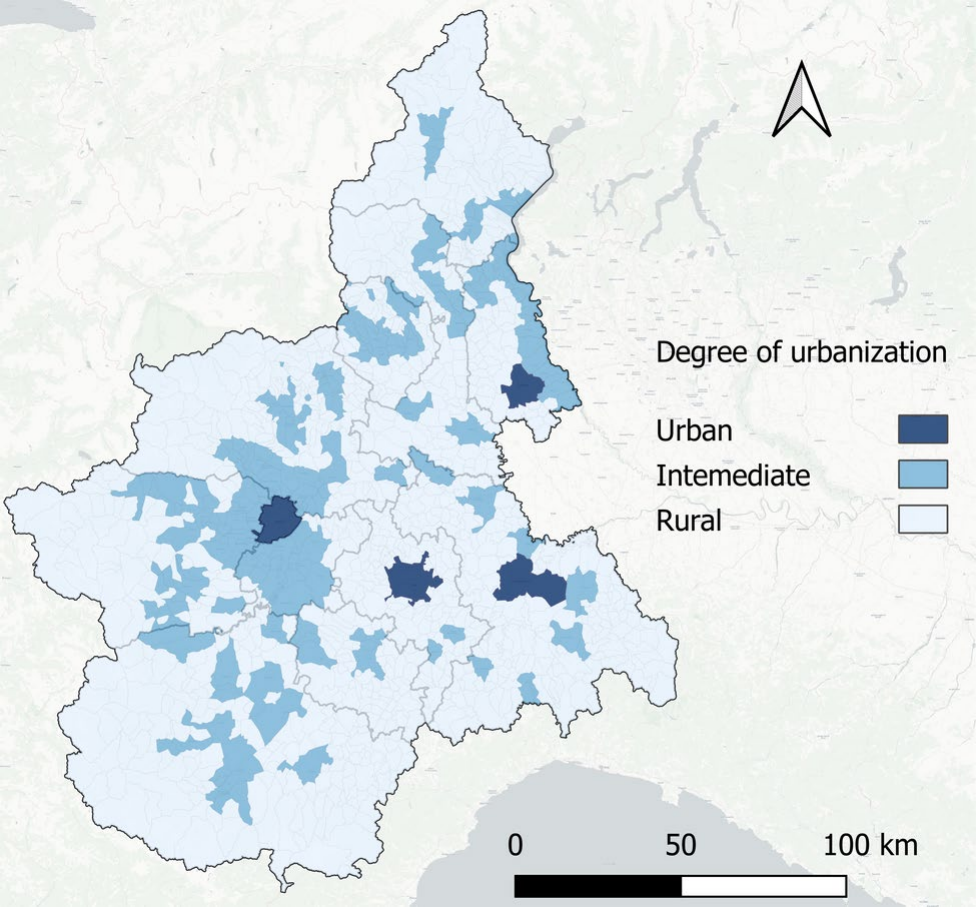
Urbanization level of the Piedmont Region

In this study, we use the 2SFCA methodology to compute accessibility in the Piedmont area. Piedmont is a large region situated in northern Italy (around 4.2 million inhabitants in 2022 according to the Italian National Statistics Institute—[ISTAT]), characterized by a combination of large and small urban centers, combined with scattered rural areas where most of the population lives. Figure 1 shows the areas with different levels of urbanization in the region, based on the Eurostat classification [16]. Only four municipalities out of 1180 are located in an urbanized area (*urban*), accounting for 4.5% of the entire region. The majority of the land in the region (71.5%) comprises *rural* areas, while the rest (24%) is classified as *intermediate*. In terms of population, around 2.1 million inhabitants (46%) live in *intermediate* areas, 29% reside in *rural* areas, and only 25% in *urban* ones.

The three main parameters of 2SFCA are population ($u_i$), service capacity ($w_j$), and distance (*d* and $d^*$). For assessing current and future accessibility at the micro-level, this study relies on population data ($P_i$) gathered from the ISTAT CENSUS database [23]. For census purposes, Piedmont is divided into 35,672 cells ($|I| = 35,672$), of which 50.3% are located in *urban* areas, 32.6% in *intermediate* areas, and 17.1% in *urban* areas.^1^ Future accessibility is based on the projections gathered from the regional demographic observatory of Piedmont, DEMOS Piemonte [14]. These projections align with national trends and indicate a gradual decrease in population, accompanied by an aging demographic composition. Specifically, the decrease in the number of inhabitants in the region varies from -0.8% to -1.6% over a period of 5 and 15 years, respectively. The impact of an aging population is more severe, with the increase in the number of elderly individuals (defined as those aged 65 and above) estimated to be +1.8%, +6.4% and +13.6% over a period of 5,10, and 15 years, respectively.

**Fig. 2:**
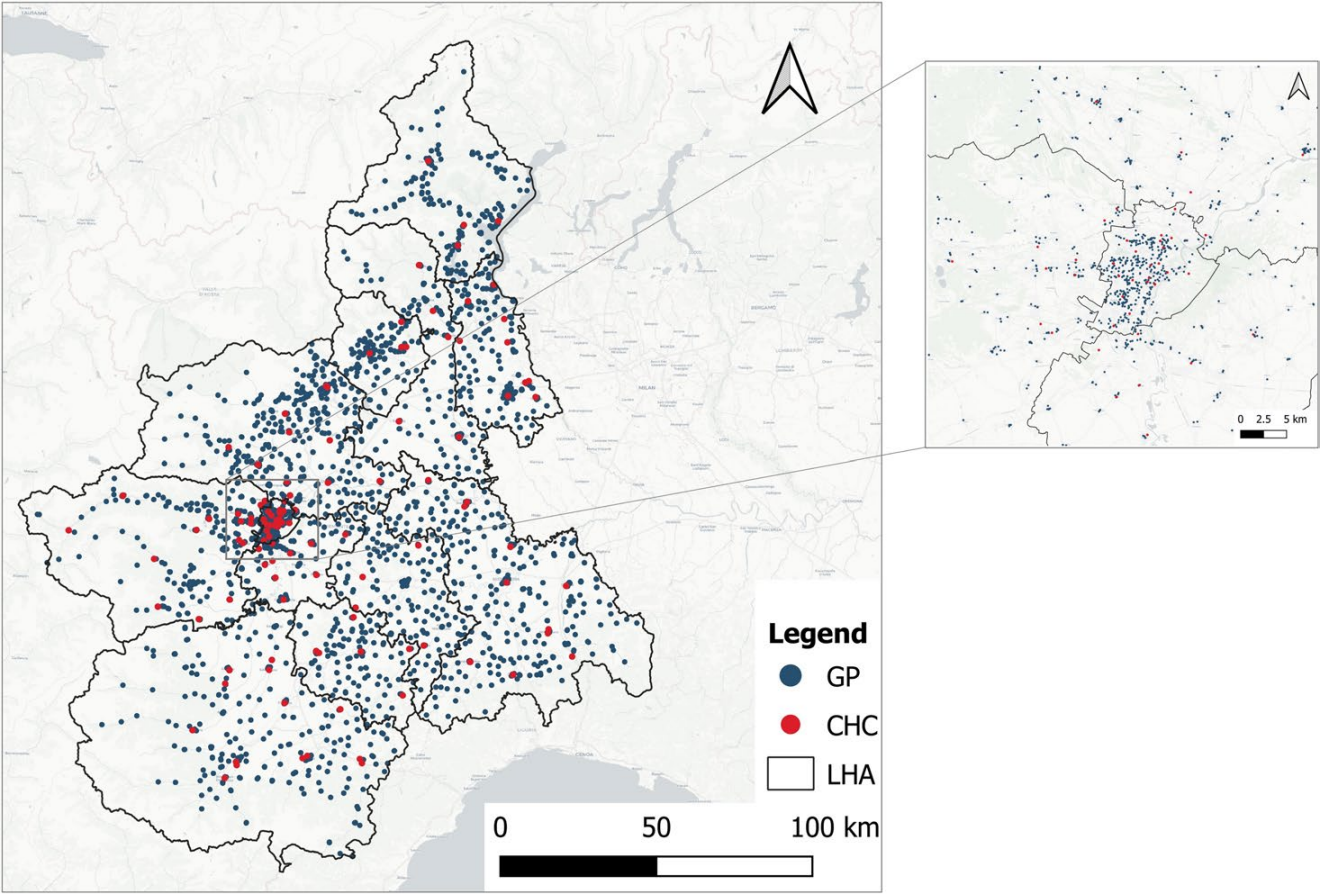
Distribution of GPs and CHCs in the Piedmont Region, with a zoom on the city of Turin

In terms of healthcare supply ($w_j$), the Piedmont region is divided into 12 Local Health Authorities (LHAs) (see Fig. 2) and accounts for 2,815 GPs spread across 2,841 different locations ($|J| = 2,841$). Supply data have been retrieved from the Piedmont healthcare regional center [43] and contain details about the hourly availability of GPs in each ambulatory. The distribution of GPs in LHAs is highly heterogeneous, with most GPs located close to two large city centers (Turin and Alessandria), while LHAs positioned in the other Piedmont provinces host only 45% of the GPs. Most of the GPs are located in intermediate areas (46%), while urban and rural areas account for around 26% and 27% of the total number of GPs, respectively. In most instances, there is no one-to-one correspondence between a specific location and the presence of a GP. Approximately 30% of the GP locations accommodate more than one GP. Equally noteworthy is the fact that approximately 38% of the GPs do not confine their services to a single location. In other words, more than 1000 GPs are distributed across multiple ambulatories.

Regarding future projections of GP availability, ISTAT historical data [25] indicates a diminishing trend in Piedmont. The average number of GPs per 10,000 people was 7.09 in 2016. This value reached its lowest in 2021, at 6.76. In absolute values, the reduction corresponds to a negative Compound Annual Growth Rate (CAGR) of -1.24% over a span of five years. This study relies on this value to project the prospective availability of primary healthcare services in Piedmont. Given the scarcity of information on the possible future variation of service at each location, we assume this decrease to be homogeneously distributed in the Region.

The assessment of future accessibility levels extends beyond the presence of GPs in the territory to account for the presence of CHCs. In line with NRRP requirements, the Piedmont region has already identified 91 CHCs located throughout the territory [41], which are expected to be fully operational by the end of 2026 (see Fig. 2). Although the CHC locations have already been designated, not all of them are currently operative, and there are no clear indications about the hours of primary healthcare services that should be offered at each site. In fact, most of these facilities require partial or complete renovation, many are still under construction, and some of them, even if structurally ready, lack medical resources.

To better account for actual GP availability and the needs of the population, this study includes some adjustments to the standard first step of the 2SFCA method (Eq. 1). Specifically, service capacity takes into account the peculiarity of the distribution of GP working hours in the different locations. $w_j$ is quantified as the sum of weekly hours during which GPs provide their services at location *j*. Furthermore, instead of solely accounting for the number of individuals residing in census cell *i*, we adopt an alternative measure to account for the fact that the primary healthcare service needs are not uniformly distributed among the population. In fact, the demand for GP visits is acknowledged to vary according to socio-demographic factors, such as age, gender, income, and education [4, 19]. According to ISTAT [24], the Italian population exhibits great heterogeneity in the frequency of GP visits, reporting statistics that vary in relation to age and gender. For example, people aged 80 and older go to the doctor about 0.8 times a month, which is more than four times the frequency of individuals aged between 25 and 35 years. Moreover, on average, the GP visit frequency of females is 30% higher than that of males. To account for these differences, we adjust the demand measure $u_i$ by considering the weighted average number of monthly visits required by the population living in cell *i*, which constitutes the demand. Specifically, for each cell *i*, we compute the total expected number of visits by summing, across all age and gender groups, the product of: (i) the visit rate (i.e., the number of visits per 100 people for that specific age-gender group), based on the most recent data available from [24], and (ii) the actual number of people in that group living in cell *i*.

Finally, along with demand and capacity, the 2SFCA model requires the identification of population-to-facility distances (*d*) and a threshold distance $d^*$, defining the catchment area boundaries. In accordance with the previous literature, which measures $d_{ij}$ as travel time or distance [3, 30, 34], $d_{ij}$ is calculated for each pair $(i,j),i\in I,j\in J$ as the shortest path on the road network, as a proxy of travel distances. As for $d^*$ there is no common measure in the literature, strongly depending on the context in which it is studied, we conduct our accessibility analysis at different thresholds, namely, $d^* \in \{1, 2.5, 5, 10\}$ km. Also, we highlight that the Italian healthcare system allows a patient to be served only by GPs that operate in the same LHA. Accordingly, $R_j$ and $A_i$ only account for the population residing in the same LHA as that of the location *j*.

## Accessibility analysis

### Estimates of current accessibility: urban-rural disparities

The 2SFCA method’s final outcome is a continuous measure of accessibility that outlines the relative difference between geographical areas (the higher the outcome, the higher the accessibility level). In practice, we have an accessibility score, say $A_i$, for each patient node $i \in I$. Overall, these $A_i$ values convey a discrete distribution of accessibility scores across the whole set of patients *I*. Hence, in order to provide a comprehensive overview of the differences in accessibility, two types of indicators are used. First, we rely on the average accessibility, that is $\overline{A}$ ($\overline{A} = \frac{\sum _{i \in I}A_i}{|I|}$). Second, we consider the fraction of potential demand $\gamma$ that is ‘‘covered’’, namely, the portion of patients whose accessibility score is at least equal to given threshold $A^\star$ (i.e., $\gamma (A^\star ) = \frac{\sum _{i \in I: A_i \ge A^\star }u_i}{\sum _{i \in I}u_i}$). Note that similar approaches have been used for distance-based accessibility evaluations (see, e.g., [7, 8]). To highlight potential territorial imbalances, the two indicators are specialized by the Degree of Urbanization (DU).

Table 1 shows the variations in the current average accessibility levels by DU across four distance thresholds ($d^*\in \{{1, 2.5, 5, 10}\}$ km). We denote these average scores by $\overline{A}_{urban}$, $\overline{A}_{intermediate}$, and $\overline{A}_{rural}$. The scores indicate lower accessibility in rural areas compared to urban and intermediate areas, with the disparity becoming more pronounced at shorter threshold distances. Considering the average accessibility level in the urban area as the reference case, at $d^*=1$ km, the rural (intermediate) areas demonstrate -57% (-18%) lesser accessibility. These differences reduce as $d^\star$ expands beyond 1 km, indicating that the average accessibility scores in intermediate and rural areas increase more than in urban areas. Still, these differences remain non-negligible. Indeed, when the threshold distance is 10 km, the rural-urban relative difference almost halves, reaching -30%. We shall note that accessibility does not always follow a monotonic trend with distance. Specifically, we observe a variation in average accessibility levels for intermediate areas, ranging from -7% at 5 km to -13% at 10 km. This can be explained by the opposing effects of $d^\star$ that reduces the supply-to-demand ratios $R_j$ and, at the same time, increases access opportunities for each user (thus, the accessibility score $A_i$). However, if the latter does not compensate for the reduction in $R_j$, accessibility scores may decline. This is precisely what occurs when increasing $d^*$ from 5 km to 10 km, as the demand within the catchment areas of health facilities in intermediate areas grows, on average, more than that in urban zones.

**Fig. 3:**
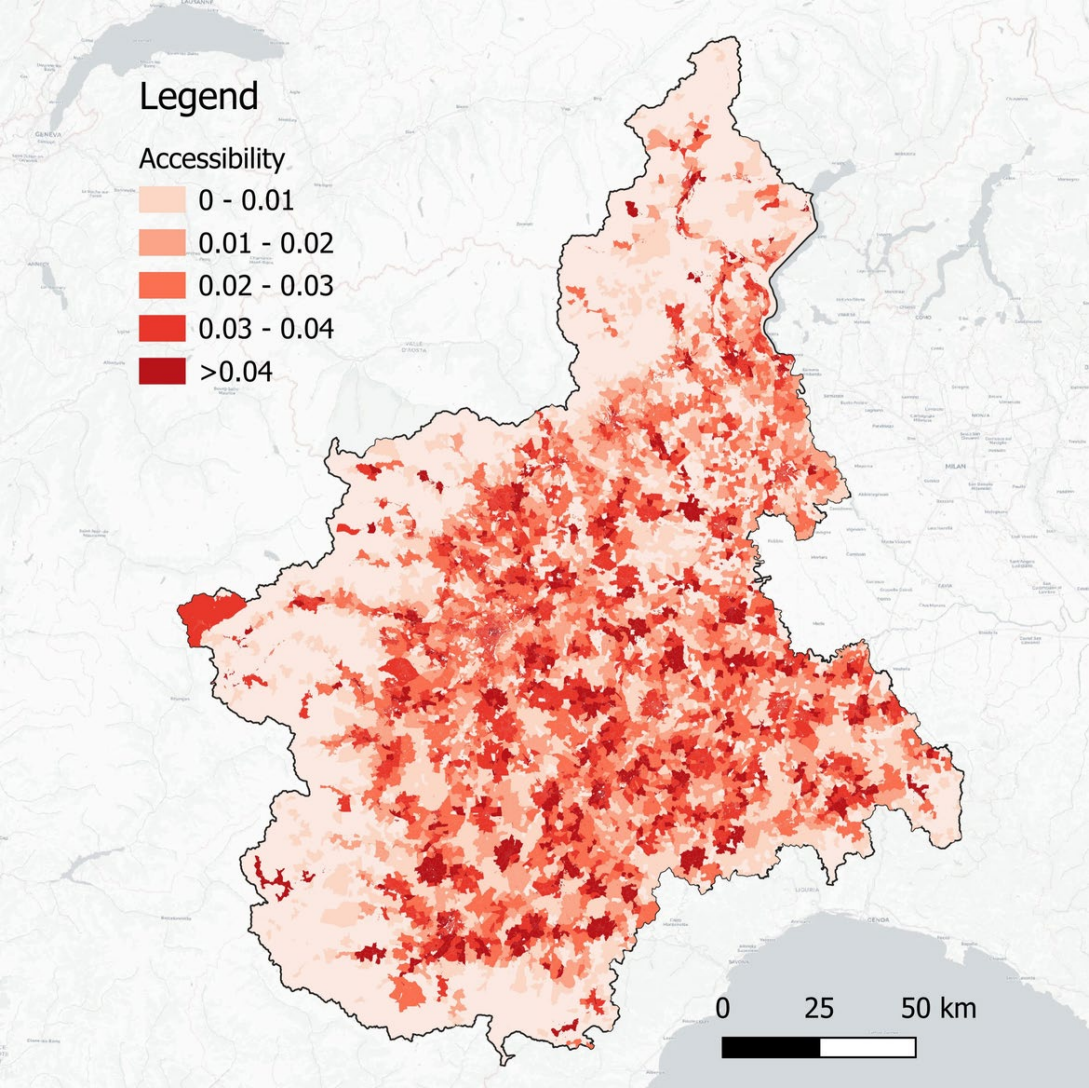
Accessibility score - darker areas indicate a higher accessibility level

For the sake of conciseness, the remainder of this paper considers the reference threshold distance as $d^* = 5$ km, in which the accessibility of rural (intermediate) areas is 30% (7%) lower than that in urban areas. Figure 3 shows the current accessibility level of each location *i* in the region.

**Table 1:** Assessment of the variation in the average accessibility levels ($\overline{A}$) by degree of urbanization (DU) across different threshold distances ($d^*$)

Threshold distance— $d^*$ (in km)				
DU	1	2.5	5	10
Urban ($\overline{A}_{urban}$)	base case	base case	base case	base case
Intermediate ($\overline{A}_{intermediate}$)	-18%	-11%	-7%	-13%
Rural ($\overline{A}_{rural}$)	-57%	-36%	-30%	-30%

**Table 2:** Assessment of the proportion of covered demand assessment at different target accessibility levels $A^*$ (threshold distance $d^*$ = 5 km)

Target accessibility values —$A^*$			
DU	$\gamma (\overline{A}_{urban})$	$\gamma (\overline{A}_{intermediate})$	$\gamma (\overline{A}_{rural})$
Urban	54%	75%	94%
Intermediate	49%	62%	86%
Rural	36%	44%	64%
Piedmont	47%	60%	82%

Assuming the average accessibility scores by DU at $d^*=$ 5 km as reference points, Table 2 illustrates the corresponding proportions of patients who have an accessibility level higher than those thresholds (i.e., $\gamma (A^\star )$, with $A^\star \in \{\overline{A}_{urban}, \overline{A}_{intermediate}, \overline{A}_{rural}\}$). Consistent with the average accessibility measure, the proportion of covered demand is lower in rural areas than in intermediate and urban zones, suggesting the presence of a great disparity among differently urbanized areas, accounting for the current capacity and demand.

To shed further light on this aspect, Table 3 presents the values of accessibility scores at given percentiles of patients, ranging from 10% to 50% (the median), and by degree of urbanization. Note that: (i) the minimum accessibility score is always zero and therefore not reported; (ii) for intermediate and rural areas, as well as for the Piedmont region, we show the relative deviations with respect to the baseline (i.e., the score in urban areas reported in the first row).

**Table 3:** Accessibility scores by percentile of patients and degree of urbanization

	Percentiles					
	10%	20%	30%	40%	50%	Avg
Urban	0.025	0.029	0.031	0.032	0.033	0.033
Intermediate	-18%	-14%	-8%	-3%	-2%	-7%
Rural	-59%	-43%	-33%	-23%	-17%	-30%
Piedmont	-31%	-18%	-12%	-5%	-3%	-16%

**Fig. 4:**
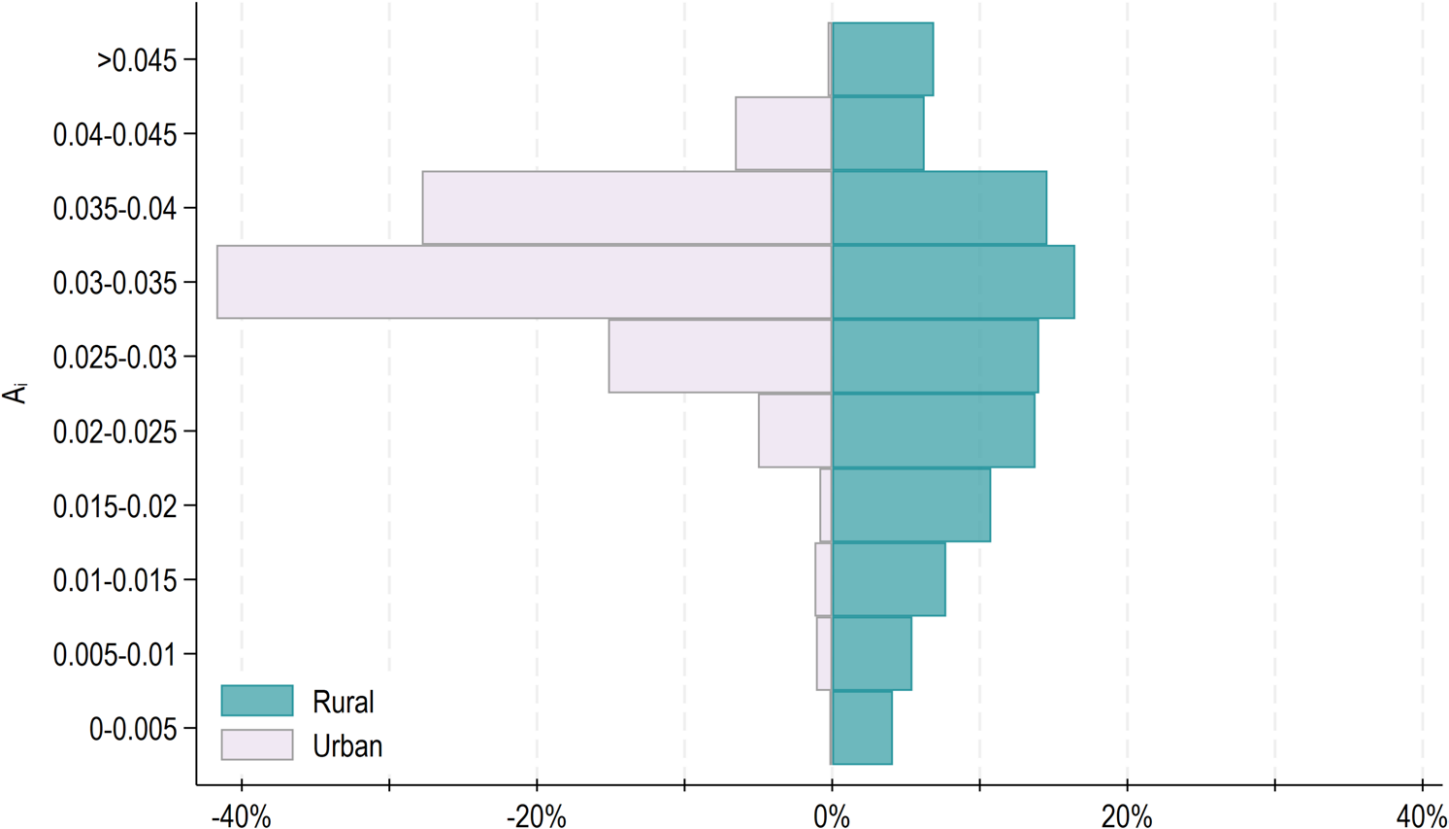
Proportion of patients by accessibility score range in urban vs. rural areas

As shown in the table, 10% of patients in urban areas have an accessibility score at most equal to 0.025. In comparison, 10% of patients in intermediate and rural areas have (at most) accessibility scores that are 18% and 59% lower than those in urban areas, respectively. These differences decrease across higher percentiles. Specifically, the median accessibility score in intermediate and rural areas is 2% and 17% lower than the baseline, respectively. The last column of the table also highlights the differences in average scores, as previously shown in Table 1. In urban areas, the average accessibility score matches the median score, whereas in intermediate and rural areas, the average score is lower than the median. This indicates that the urban distribution is more skewed toward higher values, as illustrated in Fig. 4, which displays the proportion of patients (on the *x*-axis) by accessibility score range (*y*-axis) in urban and rural areas.

In summary, the above provides a deeper investigation into the distribution of accessibility scores by degree of urbanization, emphasizing the poorer accessibility conditions in intermediate and rural areas beyond aggregate average measures.

### Future accessibility levels and regional disparities

To gauge future levels of accessibility, we replicate the 2SFCA analysis after 5, 10, and 15 years from the present ($t=2021$). By assuming $A^*=\overline{A}_{urban}$ and $d^*=$ 5 km, Table 4 summarizes the decline in average accessibility and covered demand over the years. Column 1 shows that the overall drop in average accessibility is - 7%, - 13%, and - 20% in 5, 10, and 15 years, respectively. Although the disparity among the three differently urbanized areas does not vary significantly, a slightly greater decline in accessibility can be observed in urban areas compared to intermediate and rural areas, which reduces the urban–rural accessibility gap albeit only moderately. These outcomes are consistent with the decrease in the covered demand. Column 2 of Table 4 shows the variations in covered demand over different time intervals and by degree of urbanization. In 15 years, the decrease is equal to 48 percentage points for urban areas, which is double that of the population living in rural locations (-24 percentage points). This implies that in 15 years from now, the portion of covered demand will change from 54%, 49%, and 36% to 6%, 14%, and 12% for urban, intermediate, and rural areas, respectively.

To assess the levers underpinning the decline in accessibility, we isolate the effects of three different components: population aging, population reduction, and the decrease in service capacity. Columns 3 to 5 of Table 4 illustrate the results of the deviation analysis, which account for the three effects separately.^2^

The decrease in capacity (Column 5) has the largest effect, contributing to a decrease of -14, -22, and -30 percentage points in the covered demand for the three studied time spans. Regarding the factors affecting demand, two main effects exert contradictory influences on future accessibility measures. On the one hand, population aging (Column 3) leads to a worsening of the accessibility index overall as elderly people are known to require a higher number of visits compared to younger patients [24]. On the other hand, a small decline in the number of people living in the region (Column 4) is expected to result in a small gain in accessibility. Of the two effects, population aging has the largest influence (in absolute terms), significantly contributing to the overall future decrease in accessibility. All the studied levers have a stronger impact in urban areas, which registered a decrease in the proportion of covered demand equal to -21, -35, and -48 percentage points in 5, 10, and 15 years from now.

In conclusion, these combined effects will naturally lead to a significant decline in accessibility unless service capacity is increased. CHCs provide a solution to this issue, provided they are staffed in alignment with catchment area demands over time.

Our analysis also facilitates the identification of current and future *hotspots* in terms of accessibility (e.g., census tracts with the lowest accessibility scores—see Fig. 3 for the AS-IS scenario), providing an informational basis for decision-makers to prioritize interventions in the least-served areas. Note that current and future hotspots may differ, as the impact of various levers is not uniform across different levels of urbanization. This observation adds to the complexity of the problem of redesigning GP networks and emphasizes the need for methodologies that are able to act as decision-support tools. To this end, some possible mathematical models to operate CHCs are presented in the next section.

**Table 4:** Variation in the average accessibility (1), in the proportion of covered demand (2), and levers of variation in the proportion of covered demand (Deviation Analysis) (3-5), considering $$A^*=\overline{A}_{urban}$$$$d^*=$$ 5 km

DU	(1)	(2)	(3)	(4)	(5)
	$\Delta$Average	$\Delta$Covered	Levers variation in the proportion of covered demand*		
	Accessibility	Demand*	Population Ageing	Population Reduction	GPs’ availability
In 5 years					
Urban	-7%	-21	-6	3	-18
Intermediate	-7%	-15	-3	2	-14
Rural	-6%	-9	-1	1	-9
Piedmont	-7%	-15	-3	2	-14
In 10 years					
Urban	-14%	-35	-10	5	-30
Intermediate	-13%	-27	-7	1	-21
Rural	-13%	-18	-2	1	-17
Piedmont	-13%	-26	-6	2	-22
In 15 years					
Urban	-20%	-48	-15	6	-39
Intermediate	-19%	-34	-9	3	-28
Rural	-19%	-24	-3	1	-22
Piedmont	-20%	-36	-9	3	-30

*Variation in percentage points - pp

## The role of community healthcare centers

As our findings reveal, patients’ accessibility conditions in the region are expected to worsen significantly in the mid-to-long term. In this context, the role of CHCs becomes crucial. Indeed, the presence of GPs in CHCs, as prescribed by the Italian NRRP, should provide additional opportunities for patients to receive care services, thus counterbalancing the adverse effects of population aging and the progressive decline in the number of GPs.

In this section, we assess the extent to which CHCs can help achieve this goal. In particular, to explore solutions to the current and projected levels of primary healthcare services, we look at defining the optimal availability of health resources (namely, GPs) in CHCs with a view to maximizing patient accessibility. In other words, we suppose that a decision-maker is interested in planning the allocation of GPs in CHCs to ensure that the proportion of patients provided with a minimum desirable accessibility level is maximized. Two possible strategies are explored: (i) *Strategy 1 - Capacity expansion* assumes extra availability of GPs that are assigned to CHCs, and (ii) *Strategy 2 - Capacity redistribution* is based on the re-allocation of GPs’ current capacities from their existing workplace locations to CHCs. We devise two mathematical programming models, which are presented (and whose results are reported) in the following subsections. Finally, as a hybrid version of the above options, an additional strategy— *Strategy 3*, involving both capacity expansion and redistribution decisions—is also discussed and tested. To ease the reader, the notation is listed in Appendix A—Table 7.

### Strategy 1 - Capacity expansion

Under *Strategy 1*, our aim is to optimally define the temporal (weekly) availability of new GPs that can be allocated to CHCs in order to improve the accessibility of patients. In particular, we set a target value of accessibility and try to maximize the number of patients who meet such a threshold. In line with our analysis (see Section 3), accessibility is calculated using the 2SFCA method, considering both the current and newly added availability of GPs at CHCs. We assume the total availability of GPs to be potentially allocated across the whole set of CHCs is given beforehand, i.e., it is an exogenous parameter to our problem. We also assume that GPs are not being taken away from their workplace locations for allocation elsewhere. This means, in practice, that the availability of GPs at the existing locations equals the current one, and the projected decline in this availability over time is considered. Thus, the decisions to be made pertain to the portion of this full availability that has to be allocated to CHCs. Also, as an additional feature of practical relevance, we assume that the minimum and maximum thresholds on the weekly availability of GPs at each CHC are considered.

To model the problem, some further notation is introduced as follows. Let *K* be the set of already identified locations for CHCs and $c_{ik}$ denote the distances from the patients to the CHCs. $Q_k^{min}$ and $Q_k^{max}$ are the lower and upper bounds on GPs’ availability at each location $k \in K$. Besides, $Q^{tot}$ refers to the maximum temporal availability of GPs that can be allocated to CHCs, and $A^{\star }$ refers to the target accessibility level to guarantee to the patients. Two families of decision variables are considered: (i) $q_k$, expressing the weekly availability (in hours) allocated at each CHC ($q_k \ge 0, k \in K$), and (ii) $x_i$, which is equal to 1 if patients in $i \in I$ are “covered”, i.e., their accessibility is at least equal to the envisaged target value $A^{\star }$, 0 otherwise.

Based on the above notation, the model ($M_1$), can be formulated as follows:

M1.1 $$\begin{aligned} \text{ maximize } \quad&\sum _{i \in I} u_i x_i, \end{aligned}$$

M1.2 $$\small\text{ subject } \text{ to } \quad\sum _{j \in J} \frac{w_j}{\sum _{i \in I:d_{ij} \le d^\star } u_i} + \sum _{k \in K} \frac{q_k}{\sum _{i \in I:c_{ik} \le d^\star } u_i} \ge A^{\star } x_i \qquad i \in I, $$

M1.3 $$\begin{aligned}&\sum _{k \in K} q_k \le Q^{tot}, \end{aligned}$$

M1.4 $$\begin{aligned}&Q^{min}_{k} \le q_k \le Q^{max}_{k} \qquad \qquad \quad k \in K, \end{aligned}$$

M1.5 $$\begin{aligned}&q_k \ge 0 \qquad \qquad \qquad \qquad \qquad \qquad k \in K, \end{aligned}$$

M1.6 $$\begin{aligned}&x_i \in \{0, 1\} \qquad \qquad \qquad \qquad i \in I. \end{aligned}$$

Objective function (M1.1) maximizes the covered demand, i.e., reaching the desired accessibility level. Constraints (M1.2) state that the demand in *i* is covered if its accessibility score is at least $A^\star$. Note that if the latter condition is met, the *x*-variables will be equal to 1 as objective function (M1.1) has to be maximized. Also, such an accessibility score (the left-hand side in (M1.2)) is computed as the sum of two terms: the accessibility score yielded by the actual availability of GPs in their current locations ($w_j, j \in J$, which, as assumed, is not affected by any (re-)allocation decision) and the accessibility score determined by the newly allocated availabilities of GPs at CHCs ($q_k, k \in K$). Constraints (M1.3) ensure that the total allocated temporal availability of GPs at CHCs does not exceed $Q^{tot}$, while Constraints (M1.4) guarantees that the availability of GPs at each CHC *k* is within the lower and upper bounds given by parameters $Q_k^{min}$ and $Q_k^{max}$. Finally, Constraints (M1.5)-(M1.6) define the domain of the introduced decision variables.

Model M$_1$ can be used to assess the effects of new GP allocations in the mid-to-long term. To this end, it is sufficient to use projected values for supply ($w_j$) and demand ($u_i$) parameters at a generic future time period and resolve the model accordingly. With this setting, the model yields the solution for the proportion of patients reaching the target accessibility level at the considered time period, if we ensure, by that time period, a temporal availability of GPs at CHCs that is at most equal to $Q^{tot}$.

#### Experimental tests

Model M$_1$ was tested using the Piedmont region as a case study. To this end, we need to specify some further data used (in addition to those already presented in Section 2) and the setting of the relevant model parameters, i.e., the threshold distance ($d^\star$), demand ($u_i$), supply ($w_j$), and capacity parameters ($Q^{tot}$, and, thus, $\alpha$, $Q_k^{min}$ and $Q_k^{max}$). We will detail this next.

##### Test data and parameters’ setting

We recall that the set *K* of CHC locations corresponds to the already (actual) identified sites of CHCs in the study area. The patients-to-CHCs distances $c_{ik}$ were calculated as the shortest paths on the road network. Further, the threshold distance $d^\star$, used for the computation of patients’ accessibility, was set at 5 km. Various experiments were conducted by varying the demand ($u_i$) and supply ($w_j$) parameters and using their projected values in four reference years—2021, 2026, 2031, and 2036—with 2021 denoting the AS-IS scenario. The target accessibility level $A^\star$ was set equal to the average accessibility score calculated in urban areas in the current scenario (i.e., $A^\star = \overline{A}_{urban}$, $i \in I$, in 2021), which is assumed to be a reasonably good value of accessibility. The total availability of GPs to be allocated at CHCs was set as a percentage of the total current availability of GPs, i.e., $Q^{tot} = \alpha \sum _{j \in J}w_j$, with $w_j$-values equal to those in 2021 (AS-IS) and $\alpha \in \{0.01, 0.05, 0.10, 0.20\}$. Notably, if we assume the same average weekly workload for GPs—about 14.5 hours per week in our case—we can convert hourly capacity into absolute numbers. Thus, since the current number of GPs is equal to 2,815, varying $\alpha \in \{0.01, 0.05, 0.10, 0.20\}$ would mean considering scenarios from 28 ($\alpha = 0.01$) to 563 ($\alpha = 0.20$) new GPs operating at CHCs. This observation allows for an easier interpretation of the analyzed scenarios in terms of the number of GPs involved and the associated proxied costs for their implementation. These values also align with the hiring predictions for new GPs in the region (about 500 by the end of 2024 - see https://tinyurl.com/mvtjucy5), suggesting that CHCs are capable of welcoming such capacity. The minimum and maximum availabilities of GPs at each CHC $k \in K$, i.e., $Q_k^{min}$ and $Q_k^{max}$, were set to 0 and $Q^{tot}$, respectively. This way, “capacity” restrictions at CHCs did not come into effect, and the solutions produced by the model could be used as ideal benchmarks for comparative purposes.

##### Results

All the experiments were performed on Intel(R) Core(TM) i7–8750H CPU at 2.20 GHz, equipped with 16 GB RAM and Windows 10 Pro–64 bits operating system. The optimization model was solved using the commercial solver IBM ILOG CPLEX 12.10.

**Fig. 5:**
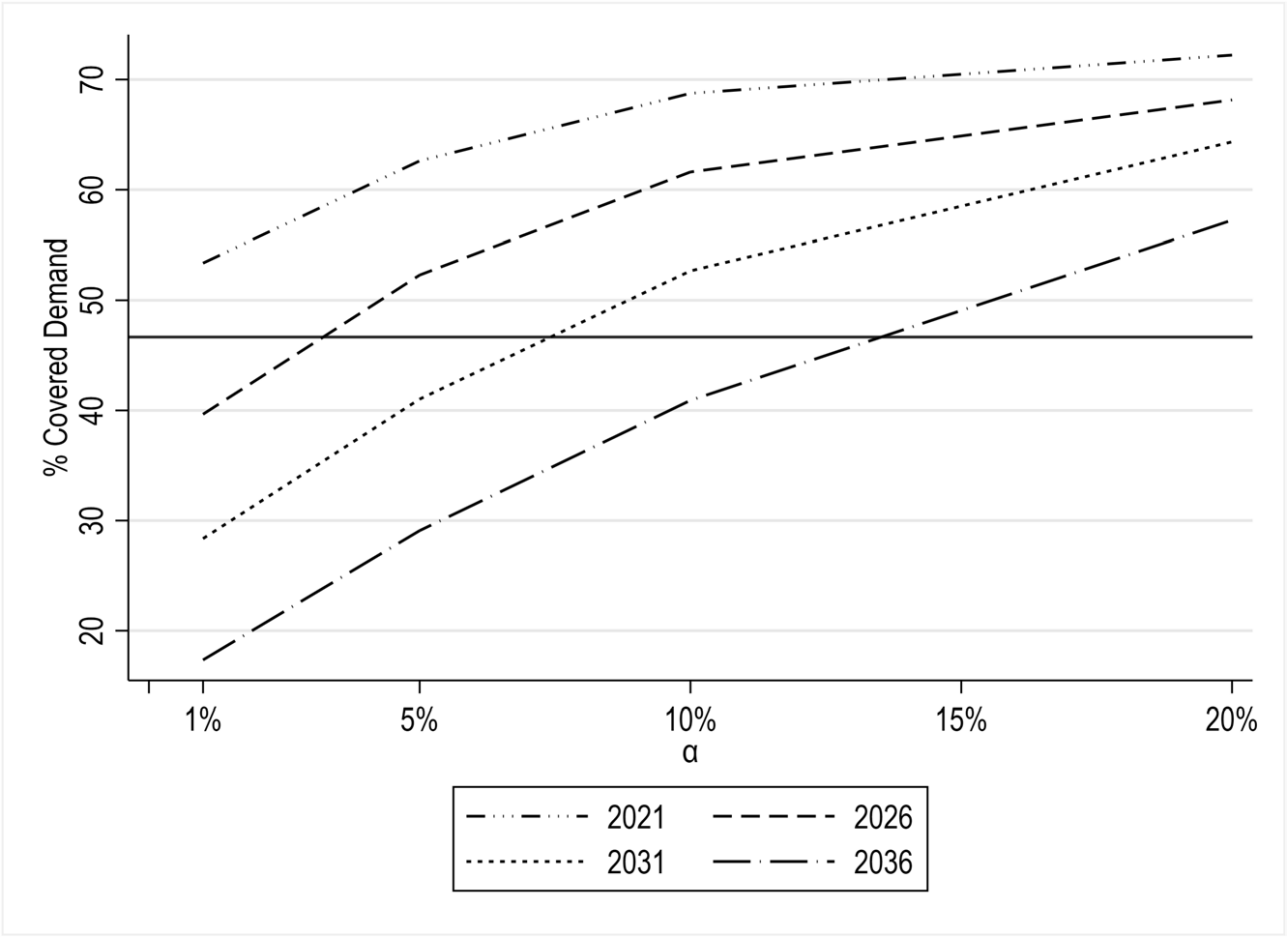
Results for model M$_1$: proportion of covered demand in the Piedmont region by $\alpha$ and time period *t* (solid black line represents the “AS-IS” values)

The obtained results are summarized in Fig. 5, which reports the proportion of demand covered (i.e., reaching the target accessibility level $A^\star$) by the value of $\alpha$ for the years 2021, 2026, 2031, and 2036. Recall that $\alpha$ regulates the total availability $Q^{tot}$ of GPs who can be allocated to CHCs. The figure also displays the percentage of covered demand in the AS-IS scenario (solid black line).

First, we focus on the results for the year 2021, where we evaluate the effects of GP allocation in the current scenario, i.e., at equal values of demand and supply parameters. Improvements in the patients’ accessibility (all other conditions being equal) are significant even for smaller values of $\alpha$. Just 1% more of GPs’ availability at CHCs (i.e., $\alpha = 0.01$ , that is approximately 28 new GPs) would increase the proportion of covered demand from 47% (AS-IS) to 53%. A higher gain (+25%) is derived when $\alpha = 0.20$, which is about 563 new GPs.

The above results change significantly when the projected values for supply and demand are considered. For $\alpha = 0.01$, the proportion of covered demand reduces to 40% in 2026. In practice, the corresponding availability of GPs at CHCs would not be sufficient to sustain the current accessibility conditions due to the expected incumbent effects of population aging and the reduction in the number of GPs at the existing locations. Such a decrease would be more severe in the long term. Note that the proportion of covered demand would be equal to 28% and 17% in 2031 and 2036, respectively.

Nevertheless, leveraging the availability of GPs at CHCs is a viable option to counterbalance these trends. Clearly, the impact of these interventions is strongly dependent on the length of the decision-maker’s planning horizon. While between 1% and 5% of the current availability of GPs would be enough to ensure (and improve) the AS-IS accessibility conditions by 2026, this value would be in the ranges [5%, 10%] and [10%, 20%] for 2031 and 2036. In other words, to prevent healthcare accessibility in the region from worsening, the availability of 28 to 140, 140 to 280, and 280 to 560 additional GPs would be needed in the projected scenarios. Besides, observe that all the displayed lines are concave. As our results reveal, the increase in regional coverage is less than proportional w.r.t. the additional capacity $\alpha$. Moreover, they verify the diminishing returns principle, as the marginal increase in coverage declines by $\alpha$ (at least, for the chosen test values).

A more in-depth evaluation is provided in Table 5, which reports, for each year and value of $\alpha$, the proportion of covered demand by degree of urbanization. In addition, the column titled “$\alpha = 0$” shows the changes in such proportions if no actions are taken, i.e., no additional GPs are made available at CHCs. Here, it is useful to recall the following conditions: (i) parameter $Q^{tot}$ indicates the maximum temporal availability of GPs that can be (re-)allocated to CHCs, and (ii) we define $Q^{tot}$ as a percentage $\alpha$ of the current weekly availability of GPs in the region. Thus, $\alpha = 0$ implies that $Q^{tot} = 0$, and this forces the *q*-variables to be 0 (see Constraints (M1.3)). As a result, the percentages found in Table 5 under the “$\alpha = 0$ column” indicate the current and projected accessibility conditions if the CHCs are not operating. Note that the latter correspond to those already shown in Section 3, which we consider as reference values to assess the contribution of CHCs. Clearly, they are equal to the AS-IS values for the 2021 scenario. The cells showing percentages higher than or equal to those in the AS-IS scenario (i.e., 54%, 49%, 36%, and 47% for urban, intermediate, rural areas, and the entire Piedmont region, respectively) are also highlighted in bold. Thus, Table 5 ultimately illustrates the total availability/number of GPs needed at CHCs to preserve or improve patients’ accessibility in each considered scenario (year).

**Table 5:** Results for *Strategy 1* (model M$_1$): proportion of covered demand by the Degree of Urbanization (DU), $\alpha$, and scenario (year)

Scenario (year)	DU		$\alpha = 0$	$\alpha = 0.01$	$\alpha = 0.05$	$\alpha = 0.10$	$\alpha = 0.20$	$\alpha \rightarrow +\infty$
2021	Urban		54%	**65%**	**77%**	**89%**	**94%**	**97%**
	Intermediate		49%	**57%**	**69%**	**76%**	**80%**	**82%**
	Rural		36%	**38%**	**39%**	**40%**	**41%**	**42%**
	Piedmont		47%	**53%**	**63%**	**69%**	**72%**	**74%**
2026	Urban		33%	45%	**68%**	**83%**	**93%**	**97%**
	Intermediate		34%	41%	**55%**	**67%**	**75%**	**77%**
	Rural		28%	32%	34%	35%	**36%**	**38%**
	Piedmont		32%	40%	**52%**	**62%**	**68%**	**71%**
2031	Urban		20%	20%	**59%**	**76%**	**90%**	**97%**
	Intermediate		23%	35%	41%	**56%**	**71%**	**75%**
	Rural		19%	26%	26%	27%	30%	32%
	Piedmont		21%	28%	41%	**53%**	**64%**	**68%**
2036	Urban		6%	6%	36%	**66%**	**79%**	**91%**
	Intermediate		14%	23%	30%	39%	**65%**	**71%**
	Rural		12%	19%	21%	22%	25%	28%
	Piedmont		12%	17%	29%	41%	**57%**	**64%**

We observe that accessibility improvements are far more noticeable in urban areas than in rural ones. This fact is consistent with objective function (M1.1), which maximizes the number of patients reaching the accessibility target, and hence is expected to produce more significant impacts in densely populated zones. However, our results also suggest that GP allocation decisions may exacerbate inequalities in the accessibility of primary care services. This is evident in the differences between the reported values for urban and rural areas in the AS-IS and future scenarios. This is possibly because the identified locations for CHCs, which are mainly distributed in urban areas, do not allow for an adequate compensation of accessibility deterioration in rural contexts. Such hypothesis is confirmed upon observing that the model always tends to achieve the highest possible proportions of demand covered in rural areas, corresponding to the percentages of patients residing within $d^\star = 5$ km from a CHC, and reported in the last column of Table 5 “$\alpha \rightarrow +\infty$”^3^). This suggests that while the role of CHCs is undoubtedly crucial to reinforce territorial medicine in the mid-to-long term, alternative solutions (e.g., identifying new locations for CHCs, allocating new GPs to the existing workplace locations) are needed if a more equitable planning of health resources is sought.

### Strategy 2 - Capacity redistribution

We now assume that reallocating GPs from their current locations to CHCs is the only viable decision available to the decision-maker to improve patients’ accessibility. In practice, for this case, we are assuming that no additional service hours (thus, no new GPs) can be allocated to the CHCs. Thus, the problem consists of identifying the optimal temporal availability of GPs at CHCs by redistributing GPs from their current locations in order to maximize the number of patients reaching the target accessibility level $A^{\star }$. The Piedmont healthcare system (like all other regional Italian systems) is divided into LHAs, to which GPs belong and where they can provide primary healthcare services. LHAs are also responsible for GPs’ service provision and are in charge of ensuring minimum assistance levels to patients. To this end, the national regulation foresees the possibility of LHAs having to evaluate and implement changes to GPs’ locations to better organize territorial care services [39]. Therefore, as an additional practical condition, we consider that GPs can only be reallocated to CHCs within the same LHA.

To model the problem, let us denote by $l_j \, [l_k]$ the LHA location *j* [CHC *k*] belongs to. Accordingly, for each original GP location $j \in J$, we denote by $N_j$ the subset of CHCs in which reallocation decisions are forbidden, i.e., those belonging to a different LHA ($N_j = \{k \in K: l_j \ne l_k\}$). We also introduce the following two decision variables: (i) $\Delta _{jk}$, indicating the amount of GPs’ availability reallocated from the original location $j \in J$ to CHC $k \in K$, and (ii) $w^\prime _j$, accounting for the residual availability of GPs at original locations $j \in J$, resulting from the redistribution decisions.

The corresponding model ($M_2$) can be formulated as follows:

M2.1 $$\begin{aligned} \text{ maximize } \quad&\sum _{i \in I} u_i x_i, \end{aligned}$$

M2.2 $$\begin{aligned} \text{ subject } \text{ to } \quad&\sum _{j \in J} \frac{w^\prime _j}{\sum _{i \in I:d_{ij} \le d^\star } u_i} + \sum _{k \in K} \frac{q_k}{\sum _{i \in I:c_{ik} \le d^\star } u_i} \\& \ge A^{\star } x_i \qquad \,\,\, i \in I, \end{aligned}$$

M2.3 $$\begin{aligned}&w^\prime _j = w_j - \sum _{k \in K} \Delta _{jk} \qquad \qquad \qquad \qquad \qquad \qquad \qquad \qquad \quad \,\,\, j \in J, \end{aligned}$$

M2.4 $$\begin{aligned}&q_k = \sum _{j \in J} \Delta _{jk} \qquad \qquad \qquad \qquad \qquad k \in K, \end{aligned}$$

M2.5 $$\begin{aligned}&\sum _{k \in K} \Delta _{jk} \le w_j \qquad \qquad \qquad \qquad \qquad \qquad \qquad \qquad \qquad \qquad \,\,\, j \in J, \end{aligned}$$

M2.6 $$\begin{aligned}&\sum _{j \in J} \sum _{k \in K} \Delta _{jk} \le Q^{tot} , \end{aligned}$$

M2.7 $$\begin{aligned}&\Delta _{jk} = 0 \qquad \qquad \qquad \qquad \qquad \qquad \qquad \qquad \qquad \quad \,\, j \in J, k \in N_j, \end{aligned}$$

M2.8 $$\begin{aligned}&x_i \in \{0, 1\} \qquad \qquad \qquad \qquad \qquad \qquad i \in I, \end{aligned}$$

M2.9 $$\begin{aligned}&q_k, w^\prime _j, \Delta _{jk} \ge 0 \qquad \qquad \qquad \qquad \qquad \qquad \qquad \qquad j \in J, k \in K. \end{aligned}$$

In the above model, objective function (M2.1) is the same as (M1.1). Constraints (M2.2) have the same meaning as inequalities (M1.2). Here, however, the first term in the LHA depends on $w^\prime _j$, i.e., the final availability of GPs at their original locations $j \in J$. The latter is calculated, for each location $j \in J$, by subtracting from the original availability $w_j$ the total amount transferred to CHCs (i.e., $\sum _{k \in K}\Delta _{jk}$), as stated by Equalities (M2.3). Variables $q_k$, already introduced for model M$_1$, still denote the availability of GPs at CHCs $k \in K$, which are now obtained as the sum of the amounts redistributed from the original locations $j \in J$ (i.e., $\sum _{j \in J}\Delta _{jk}$, see Equalities (M2.4)). Constraints (M2.5) are logical conditions expressing that the total amount of GP availability transferred from each location *j* to CHCs cannot exceed the initial availability therein $w_j$. Constraints (M2.6) define the maximum hourly availability $Q^{tot}$ of GPs that can be reallocated to CHCs. Constraints (M2.7) forbid GPs’ reallocation from existing locations *j* to CHCs not belonging to the same LHA. Finally, Constraints (M2.8)–(M2.9) give the domains of the decision variables.

We also remark that model M$_2$ can be used to assess the effects of GPs’ reallocation in the mid-to-long term. In practice, the same considerations as model M$_1$ apply here (see the end of Section 4.1) as it is sufficient to use projected values for both supply ($w_j$) and demand ($u_i$) and resolve the model accordingly. This way, model M$_2$ yields the proportion of demand reaching the target accessibility level at a given future time period if we ensure, by that time period, a temporal availability of GPs—redistributed from original locations *J*—equal to $\sum _{k \in K} q_k = \sum _{j \in J}\sum _{k \in K} \Delta _{jk}$.

#### Results and comparison between the two strategies

We implement model M$_2$ by replicating the same experimental setting as for model M$_1$ in terms of the test values used for supply, demand, target accessibility, and values of $Q^{tot}$. For the latter, an $\alpha$-percentage of the current availability is again considered, with $\alpha \in \{0.01, 0.05, 0.10, 0.20\}$. In addition, as with *Strategy 1*, an experiment with a very high value of $\alpha$ is also performed ($\alpha \rightarrow + \infty$)^4^.

Table 6 shows the obtained results for the proportions of covered demand by the value of $\alpha$ and degree of urbanization (DU). We also compare them with those obtained using *Strategy 1* (i.e., model M$_1$). Besides, as in Table 5, cells showing higher percentages than the AS-IS ones are in bold. For clarity, it is worth highlighting again that $\alpha = 0$ means that no (re-)allocation decisions are made. Indeed, given our definition of $Q^{tot}$, $\alpha = 0$ forces $\Delta$-variables to equal 0 (see Constraints (M2.6) in model M$_2$). As a result, the percentages found in Table 5 under the “$\alpha = 0$ column” must be the same for both strategies as they indicate the current and projected accessibility conditions if CHCs are not operating. A similar discussion is provided for *Strategy* 1 in Section 4.1.1—under the “Results” subsection.

**Table 6:** Comparison between Strategies 1 and 2 (models M$_1$ and M$_2$): Proportion of covered demand by the degree of brbanization (DU), $\alpha$, and scenario (year)

DU	Scenario (year)	Strategy	$\alpha = 0$	$\alpha = 0.01$	$\alpha = 0.05$	$\alpha = 0.10$	$\alpha = 0.20$	$\alpha \rightarrow + \infty$
Urban	2021	1	54%	**65%**	**78%**	**89%**	**94%**	**97%**
		2	54%	**61%**	**67%**	**72%**	**77%**	**89%**
	2026	1	33%	45%	**68%**	**83%**	**93%**	**97%**
		2	33%	42%	50%	**60%**	**67%**	**82%**
	2031	1	20%	20%	**59%**	**76%**	**90%**	**97%**
		2	20%	20%	34%	42%	**68%**	**83%**
	2036	1	6%	6%	36%	**66%**	**79%**	**91%**
		2	6%	6%	13%	30%	53%	**59%**
Intermediate	2021	1	49%	**57%**	**69%**	**76%**	**80%**	**82%**
		2	49%	**58%**	**70%**	**74%**	**74%**	**78%**
	2026	1	34%	41%	**55%**	**67%**	**75%**	**77%**
		2	34%	43%	**59%**	**67%**	**69%**	**71%**
	2031	1	23%	35%	41%	**56%**	**71%**	**75%**
		2	23%	35%	49%	**59%**	**65%**	**71%**
	2036	1	14%	23%	30%	39%	**65%**	**71%**
		2	14%	22%	39%	**50%**	**63%**	**69%**
Rural	2021	1	36%	**38%**	**39%**	**40%**	**41%**	**42%**
		2	36%	**38%**	**39%**	**39%**	**40%**	**40%**
	2026	1	28%	32%	34%	35%	**36%**	**38%**
		2	28%	32%	34%	35%	35%	35%
	2031	1	19%	26%	26%	27%	30%	32%
		2	19%	26%	27%	28%	29%	30%
	2036	1	12%	19%	21%	22%	25%	28%
		2	12%	19%	22%	23%	24%	25%
Piedmont	2021	1	47%	**53%**	**63%**	**69%**	**72%**	**74%**
		2	47%	**53%**	**60%**	**63%**	**65%**	**70%**
	2026	1	32%	40%	**52%**	**62%**	**68%**	**71%**
		2	32%	40%	**49%**	**56%**	**59%**	**63%**
	2031	1	21%	28%	41%	**53%**	**64%**	**68%**
		2	21%	28%	39%	46%	**55%**	**62%**
	2036	1	12%	17%	29%	41%	**57%**	**64%**
		2	12%	17%	27%	37%	**49%**	**54%**

The interpretation of the results from *Strategy 2* follows that given for *Strategy 1*; therefore, for the sake of brevity, we omit this discussion and focus only on a comparative assessment of the two strategies. The main evidence we observe is that *Strategy 2* performs reasonably well against *Strategy 1* only in the case of reduced availability (number) of GPs (i.e., for small values of $\alpha$). Indeed, the performance gap between the strategies is low only for the smallest tested value of $\alpha$ (0.01). Gaps increase with $\alpha$, and larger differences are observed in urban and intermediate areas, as well as in the overall Piedmont region, especially in future scenarios. In practice, as the percentage of GPs reallocated increases, the improvements in accessibility for patients living close to CHCs do not adequately compensate for the losses in the proximity of GPs’ original workplace locations. These results highlight the limitations of sole reallocation decisions, underscoring the need for hiring policies if certain accessibility levels (the AS-IS ones, in our case) must be maintained or even improved.

**Fig. 6:**
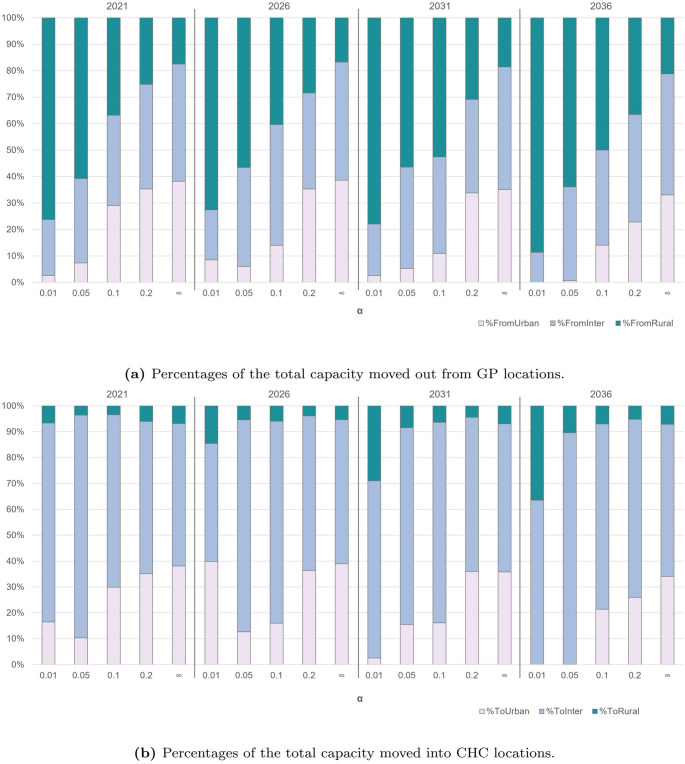
Analysis of capacity transfers in the solutions produced by *Strategy 2* (model M$_2$) across urban, intermediate, and rural areas

Nevertheless, if we solely focus on *Strategy 2*, its performance is generally positive, as it would still lead to significant improvements in the projected future accessibility (see the “$\alpha = 0$” column) for all the tested values of $\alpha$. This signifies that GP reallocation decisions are effective for the envisaged objective. To better understand this outcome, we now perform an in-depth examination of reallocations in the produced solutions. Specifically, this additional analysis aims to assess (i) where GPs’ availability is transferred from, i.e., how much percentage of it comes from urban, intermediate, and rural areas, and (ii) where such availability is transferred to, namely, how much percentage of it is reallocated to CHCs in urban, intermediate, and rural areas. The results are summarized in Figs. 6a and b, respectively.

For each solution (i.e., a combination of year and $\alpha$), Fig. 6a depicts how much of the total capacity transferred ($\alpha$% of the current availability of GPs) comes from existing workplace locations in different urbanized areas. As an example, we observe that for the year 2021 and $\alpha = 0.01$, the corresponding reallocated capacity is mainly made up of GPs operating in rural areas (76.21%—the green bar) and in smaller fractions by those operating in intermediate (21.21%—the light blue bar) and urban areas (2.58%—the white bar). As $\alpha$ increases, these percentages tend to balance, suggesting that as long as small values of $\alpha$ are considered, it is preferable to move resources from rural areas. Reasonably, if only a reduced number of GPs can be reallocated, moving them from less to more densely populated areas promotes improvements in the overall accessibility conditions. However, this is not always the case. Figure 6b shows the percentages of the total transferred capacity reallocated to (CHCs located in) different urbanized areas. Regardless of the year, we generally notice that the model tends to reallocate GPs’ availability in urban and intermediate-area facilities, where most of the population and most CHCs are located. Only in 2036, and for smaller values of $\alpha$ (0.01 and 0.05), we do not see a reallocation toward urban areas. Instead, higher percentage reallocations are seen for rural areas (bars 16 and 17, in green). In general, accessibility deteriorates significantly across the entire region in the long run (i.e., by 2036), particularly in urban zones (discussed in Section 3.2). When only a small number of GPs can be reallocated (i.e., lower values of $\alpha$), *Strategy 2* suggests avoiding their redistribution to urban CHCs. In urban areas, a larger number of patients live within shorter distances from CHCs, thus making the impact of a limited number of reallocated GPs more pronounced in rural and intermediate areas. In other words, achieving the same improvement in the overall regional accessibility would require reallocating a much higher number of GPs to urban CHCs to counterbalance their expected shortages (-39 percentage points by 2036—see Table 4, column 5).

In particular, the following aspect emerges as relevant to our analysis. For each solution, a comparison of the percentages of GPs moved from rural areas (Fig. 6a) and redirected towards them (Fig. 6b) shows a strongly negative “balance”: the “outgoing flows” of GPs’ availability are far higher than the “incoming” ones. Despite this, accessibility improves in rural areas w.r.t. the AS-IS conditions in 2021 and w.r.t. to the projected conditions (i.e., those reported under the $\alpha = 0$ column in Table 6 for subsequent years). These findings indicate that *Strategy 2* points to more effective use of resources: it highlights that the reallocation of GPs provides little to no contribution to accessibility of CHCs in rural areas, and thus motivates significant accessibility improvements. The results for urban and intermediate areas and the overall region follow the same reasoning, supporting the idea that generalized improvements are achievable if health resources are pooled where they are most needed. In practice, *Strategy 2* represents scenarios characterized by a high concentration of GPs—–from many and more geographically dispersed locations to fewer and less geographically dispersed CHCs. However, this geographical concentration favors better resource utilization and better accessibility, especially w.r.t. to projected conditions.

In conclusion, notwithstanding the absence of explicit economic considerations, the proposed strategies (expansion vs. redistribution) offer valuable insights for decision-makers to assess alternative future scenarios of territorial primary care, given two conflicting objectives: (i) the (expected) gains in patients’ accessibility against (ii) the corresponding (proxied) costs of GPs’ (re-)allocation. Our evidence highlights the presence of significant trade-offs; for instance, for the year 2021 in the Piedmont region (see Table 6), it would be feasible to address approximately 65% to 72% of the total regional demand by reallocating 20% (*Strategy 2*) of the current hourly availability of GPs or alternatively by allocating this additional portion specifically to CHCs (*Strategy 1*). Similar considerations apply to the other cases. Of course, the proposed models yield two extreme solutions, and more moderate versions could be obtained through “hybrid” strategies involving both allocation and reallocation decisions, as we argue next.^5^

### Strategy 3 - Hybridizing capacity expansion and redistribution decisions

As the last step of our analysis, we assume the decision-maker can combine capacity expansion and redistribution elements to optimize healthcare accessibility. In practice, we devise a “hybrid” strategy where GPs’ capacity allocation and reallocation decisions are simultaneously in play. To this end, a simple idea is to consider that the decision-maker can define beforehand how much of GPs’ availability at CHCs should result from each of the two types of decisions (allocation vs. reallocation). In modeling terms, given the total temporal availability $Q^{tot}$ to be made available at CHCs, we posit that a predefined percentage of it, say $\beta$, can result from new allocation of GPs decisions and the rest—$(1-\beta ) \times Q^{tot}$—from reallocations of GPs. This way, $\beta$ serves as a weighing factor to account for the number of GPs and the costs related to allocation and reallocation decisions (following the interpretation given for parameter $\alpha$ in Section 4.1.1).

Based on these assumptions, the corresponding model for *Strategy 3*, which we call ($M_3$), can be easily derived from model M$_2$ as follows:

M3.1 $$\begin{aligned} \text{ maximize } \quad&\sum _{i \in I} u_i x_i, \end{aligned}$$

M3.2 $$\begin{aligned} \text{ subject } \text{ to } \quad&\sum _{j \in J} \frac{w^\prime _j}{\sum _{i \in I:d_{ij} \le d^\star } u_i} + \sum _{k \in K} \frac{q_k}{\sum _{i \in I:c_{ik} \le d^\star } u_i} \\& \ge A^{\star } x_i \qquad i \in I, \end{aligned}$$

M3.3 $$\begin{aligned}&w^\prime _j = w_j - \sum _{k \in K} \Delta _{jk} \qquad \qquad \qquad \qquad \qquad \qquad \qquad \qquad \quad \,\, j \in J, \end{aligned}$$

M3.4 $$\begin{aligned}&q_k = \sum _{j \in J} \Delta _{jk} + q^{\prime }_{k} \qquad \qquad \qquad \qquad \qquad \qquad \qquad \qquad \qquad k \in K, \end{aligned}$$

M3.5 $$\begin{aligned}&\sum _{k \in K} \Delta _{jk} \le w_j \qquad \qquad \qquad \qquad \qquad \qquad \qquad \qquad \qquad \qquad \,\, j \in J, \end{aligned}$$

M3.6 $$\begin{aligned}&\sum _{k \in K} q^\prime _k \le \beta \times Q^{tot}, \end{aligned}$$

M3.7 $$\begin{aligned}&\sum _{j \in J} \sum _{k \in K} \Delta _{jk} \le (1 - \beta ) \times Q^{tot}, \end{aligned}$$

M3.8 $$\begin{aligned}&x_i \in \{0, 1\} \qquad \qquad \qquad \qquad \qquad \qquad \qquad \qquad i \in I, \end{aligned}$$

M3.9 $$\begin{aligned}&q_k, q^\prime _k, w^\prime _j, \Delta _{jk} \ge 0 \qquad \qquad \qquad \qquad \qquad \qquad \qquad j \in J, k \in K. \end{aligned}$$

In the above model, decision variables $q_k$ still denote the availability of GPs at CHC $k \in K$. Note that such quantity is now obtained, according to Constraints (M3.4), as the sum of the total availability reallocated in *k* from GPs’ existing locations ($\sum _{j \in J} \Delta _{jk}$) and the new GPs’ availability allocated in *k* (represented by decision variables $q^\prime _k$). Constraint (M3.6) guarantees that the total new availability of GPs that can be allocated to CHCs is at most equal to $\beta \times Q^{tot}$. Similarly, Constraint (M3.7) states that the availability of GPs that can be reallocated to CHCs cannot exceed $(1 - \beta ) \times Q^{tot}$. Observe that the combination of (M3.6) and (M3.7) implies that the total availability of GPs at CHCs is bounded by $Q^{tot}$ (i.e., $\sum _{j \in J} \sum _{k \in K} \Delta _{jk} + \sum _{k \in K} q^\prime _k \le Q^{tot}$). Finally, Constraints (M3.9) define the domain of the decision variables (including the $q^\prime$-variables). For completeness, we underline that objective function (M3.1) and Constraints (M3.2)–(M3.3), (M3.5), (M3.8) have been already defined for previous models. Hence, their explanation is omitted.

Two main aspects related to the present model need to be emphasized.

First, we observe that models M$_1$ and M$_2$ can be obtained as particular cases of model M$_3$ or, in other terms, that model M$_3$ generalizes the former. Indeed, if $\beta = 0$ (i.e., the decision-maker does not wish to perform new allocations), all the $q^\prime$-variables must be equal to 0 due to Constraint (M3.6). Accordingly, GPs’ availability at CHCs is only obtainable via reallocation decisions. This also makes Constraints (M3.4) and (M3.7) equivalent to (M2.4) and (M2.6), respectively. Hence, we fall into *Strategy 2* as model M$_3$ reduces, in fact, to model M$_2$. On the contrary, if $\beta = 1$ (i.e., the decision-maker does not wish to perform reallocations), all the $\Delta$-variables are null due to Constraint (M3.7). In turn, according to (M3.4), variables *q* and $q^\prime$ coincide, which implies that the final availability of GPs at CHCs is only due to new allocations. Under these circumstances, it is straightforward to see that model M$_3$ reduces to model M$_1$, and we therefore obtain *Strategy 1* as a particular case of *Strategy 3*.

Second, we emphasize that each value of $\beta \in [0,1]$ leads to an intermediate solution between that yielded by *Strategy 1* ($\beta = 1$) and *Strategy 2* ($\beta = 0$). In particular, by increasing $\beta$, we expect solutions with higher demand coverage as additional capacity allocation is less bounded (and fewer resources are reallocated from existing locations).

**Fig. 7:**
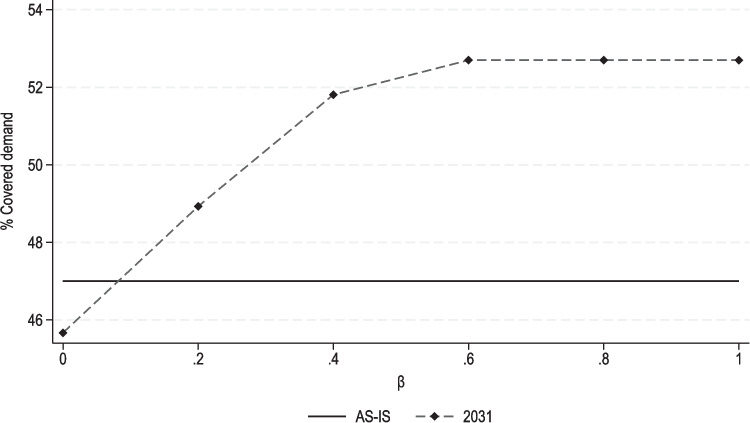
Results for model M$_3$: Proportion of covered demand in the Piedmont region by $\beta$, for the year 2031 and $\alpha = 0.10$ (solid line represents the “AS-IS” values)

To empirically demonstrate this fact, we tested model M$_3$ for each scenario (year); $\alpha = 0.05, 0.10$, and 0.20; and by varying $\beta \in [0,1]$ with a pace equal to 0.20. For brevity, we hereafter report only one case, namely, that corresponding to year 2031 and $\alpha = 0.10$. Figure 7 shows the proportions of covered demand in the Piedmont region produced by $\beta$ (dashed line). For comparison, the figure also displays the proportion of covered demand in the AS-IS scenario (2021), which is equal to 47% (solid line).

As expected, solutions to model M$_3$ yield higher demand coverage as $\beta$ increases. In particular, the proportion of covered demand equals almost 46% for $\beta = 0$ and about 53% for $\beta = 1$. Of course, the same values have been obtained by Strategies 2 and 1, as reported in Table 6. Note that, for $\beta = 0$ — i.e., *Strategy 2* — the AS-IS accessibility conditions are not maintained in the 2031 scenario. In practice, reallocating about 280 GPs (recall that $\alpha = 0.10$ in the analyzed example) would not be sufficient to prevent accessibility from worsening in 2031. However, in this case, a mix of reallocation and allocation decisions can lead to accessibility improvements. Indeed, for $\beta = 0.20$, the proportion of covered demand increases up to 49%. This means that only 56 new GPs should be allocated to CHCs, while the rest (224 GPs) should be reallocated. In addition, we also see that demand coverage reaches its maximum value for $\beta = 0.60$, i.e., if only 60% of the total availability (number) of GPs is newly allocated to CHCs. Similar evidence is found for the other test cases. In our view, these findings underscore the relevance of the proposed hybrid strategy, showcasing its ability to support the decision-making process by identifying solutions that—by leveraging both capacity expansion and redistribution actions—either approximate current accessibility conditions or maximize them with reduced staffing efforts.

## Discussion and conclusions

Motivated by the goal of improving primary care, this paper pursues two main objectives. First, we evaluate the current and future accessibility of primary care networks in an Italian region. Second, considering the introduction of new operating facilities, we propose alternative scenarios and strategies to optimize the use of these facilities (CHCs) and assess their potential impact on accessibility in the mid-to-long term. Accordingly, we estimated patients’ accessibility conditions via a tailored 2SFCA method, relying on census-level data and detailed information on GPs (i.e., workplace locations and weekly schedules/working hours). Besides, we extended this analysis to mid- to long-term scenarios using projected trends at both demand and supply levels. Then, in line with NRPP requirements, we investigated two possible strategies to maximize patients’ accessibility through CHCs: one involving the allocation of additional service hours of GPs at these facilities and the other involving their redistribution from existing workplace locations. In addition, a third strategy, which combines both allocation and redistribution decisions, has also been devised. To this end, tailored mathematical models are developed and implemented.

The primary goal of the study was to explore the feasibility of different staffing strategies in light of potential urban-rural imbalances, thus demonstrating their reasonableness based on resource availability. As such, the paper’s contributions and intent are primarily strategic in nature rather than operational or focused on direct scheduling or resource allocation. Based on these grounds, the performed strategic analysis provides the following implications for practitioners, especially in cases where future resource constraints and intra-regional disparities need to be anticipated and mitigated (like that at hand). First, the estimates on accessibility conditions at the census tract level in the region allow for the identification of the *hotspots*, i.e., the current and future areas where interventions are most needed. Second, we use future estimates of both supply and demand to demonstrate the relevance and urgency of strategic actions to reinforce territorial medicine, thus empirically supporting the need to resort to CHCs as additional primary care facilities. Third, through a relatively extensive experimental analysis, we show that CHCs are necessary to improve future patients’ accessibility over the projected scenarios (in most cases, to a considerable extent). This applies even when only capacity redistribution is considered (*Strategy 2*) as a result of more effective utilization of resources. We also note that such improvements would often be sufficient to preserve the current accessibility levels. Fourth, on the basis of the implementation of the devised mathematical models, we compute the lower and upper bounds in terms of the accessibility achievable by the two “pure” implemented strategies: new GP allocation decisions (*Strategy 1 - Capacity expansion*) and only reallocation of GPs (*Strategy 2 - Capacity redistribution*). Estimations of current and future potential accessibility conditions provide insights into the existing trade-off between allocating new capacity in CHCs and leveraging the current sample of GPs to be (partially) relocated into CHCs. Besides, the implementation of the so-called hybrid strategy (i.e., *Strategy 3*) shows that adequately combining capacity expansion and redistribution actions may yield organizational solutions that maximize regional accessibility while limiting new GP staffing. Finally, we observe that operating CHCs might potentially increase the disparities in accessibility between urban and rural areas due to the location decisions taken by the regional authorities. While they are essential, the currently identified CHCs are insufficient to attain equity in patients’ accessibility. This underscores the need for exploring alternative strategies or solutions to realize the policy objective of achieving equity.

The limitations and insights from our study present several opportunities for further developments in future research.

First, our future estimations of accessibility do not account for varying demand needs, which may depend, among others, on the level of use of digital technologies. The advancements in digitalization, exemplified by telemedicine, offer numerous advantages to the healthcare sector, especially in terms of accessibility. The adoption of telemedicine for remote visits has the potential to bridge the physical gap between patients and GPs, thus transforming the delivery of primary healthcare services. Although telemedicine is not extensively utilized for such purposes at present, its future potential is promising and significant. Consequently, future research should take this aspect into account, addressing the different needs of patients who still prefer to physically visit their GPs in ambulatories versus the needs that can be addressed via a virtual meeting. After an assessment of the market penetration of telemedicine for such purposes in the following years, adjustments should be made to the demand estimates as well as to accessibility metrics. Similarly, our estimations do not account for expected distributional changes in the population across urban and rural areas. Future endeavors should also target this limitation by assessing how ongoing rural-to-urban migration trends may impact accessibility.

Second, the proposed models could be refined to represent more specific strategies for operating CHCs. Indeed, the goal of this paper is to broadly evaluate the potential of CHCs in the regional network, and our findings are not intended to serve as prescriptive, directly implementable solutions. While various assumptions limit their sufficiency and applicability, they do offer empirical grounds and ideal benchmarks for evaluating the potential effects of the envisaged territorial reorganization of primary care. This paves the way for further, more sophisticated exploration, including the utilization of more granular data that can offer more operation-specific directions.

In addition, the revealed trade-off between increasing the workforce (i.e., hiring new healthcare professionals—*Strategy 1*) and reallocating GPs’ capacity to CHCs (*Strategy 2*), along with its impact on accessibility, calls for a comprehensive evaluation of the associated costs and benefits of the two distinct strategies [6]. While our results support strategic decision-making by revealing the potential effects of the stratgies on accessibility, assessing their economic sustainability (i.e., affordability and actual applicability) and welfare implications require further investigation.

Finally, it may also be worthwhile to undertake a comparative analysis by extending our investigation to multiple regional case studies. Indeed, given the generality of our methodological approach (i.e., the 2SFCA method and the strategies/models), we consider the extension of our approach to other regional Italian cases—or even to countries with similar organizational models—as a promising research direction to compare our findings and possibly frame them within the territorial and structural features of the specific health system at hand.

## Data Availability

Data utilized in the study are publicly available and all sources are properly cited in the manuscript.

## Appendix A: Notation

Table 7 reports the mathematical notation used in the manuscript.

**Table 7:** Mathematical notation (Note: GPs—General Practitioners; CHCs—Community Healthcare Centers; LHA—Local Health Authority)

Notation	Description
*Sets*	
*I*	set of patients’ nodes, indexed by *i*
*J*	set of existing GPs’ workplace locations, indexed by *j*
*K*	set of CHCs locations, indexed by *k*
*General parameters*	
$u_i$	demand of doctor visits in node $i \in I$
$w_j$	GPs’ availability at location $j \in J$
$d_{ij}$	distance from patient location $i \in I$ to location $j \in J$
$l_i, l_j, l_k$	LHA of patient *i*, location *j*, and CHC *k*
$c_{ik}$	distance from patient location $i \in I$ to CHC $k \in K$
$d^\star$	threshold distance defining the size of the catchment areas
$R_j$	supply-to-demand ratio at location $j \in J$
$A_i$	accessibility score of node $i \in I$
$\overline{A}$	average accessibility score
$A^\star$	accessibility threshold
$\gamma (A^\star )$	fraction of patients with accessibility at least equal to $A^\star$
*Strategies-related sets and parameters*	
$N_j$	set of CHCs to which reallocations from location *j* are forbidden (*Strategies 2 and 3*)
$Q_k^{min}$	minimum GPs’ availability to be allocated at CHC $k \in K$ (*Strategy 1*)
$Q_k^{max}$	maximum GPs’ availability that can be allocated at CHC $k \in K$ (*Strategy 1*)
$Q^{tot}$	maximum GPs’ availability that can be allocated to CHCs (*All Strategies*)
$\alpha$	fraction of GPs’ availability that can be allocated to CHCs (*All Strategies*)
$\beta$	percentage of $Q^{tot}$ resulting from new allocations (*Strategy 3*)
*Decision variables*	
$x_i \in \{0,1\}$	equal to 1 if patient node *i* is “covered”, i.e., reaches accessibility $A^\star$, 0 otherwise (*All Strategies*)
$q_k \ge 0$	GPs’ availability at CHC $k \in K$ *(All Strategies)*
$\Delta _{jk} \ge 0$	GPs’ availability reallocated from location $j \in J$ to CHC $k \in N_j$ (*Strategies 2 and 3*)
$w^\prime _{j} \ge 0$	residual GPs’ availability at location $j \in J$ following reallocation decisions (*Strategies 2 and 3)*
$q^\prime _k \ge 0$	GPs’ availability at CHC $k \in K$ resulting from allocation decisions (*Strategy 3*)

## Footnotes

1 The latest available census data on Piedmont’s inhabitants are from 2011. We adjusted the population level in year *t* in each cell *i*, relying on recent, aggregated population data gathered from DEMOS Piemonte [14]. Specifically, the number of individuals of a certain age class *a* and given gender *s* living in cell *i* at time *t* ($P_{i,t,a,s}$) is equal to $\frac{P_{i,2011,a,s}}{P_{y_i,2011,a,s}}\cdot P_{y_i,t,a,s}$, where $P_{i,2011,a,s}$ ($P_{y_i,2011,a,s}$) stands for the number of individuals of a certain age class *a* and given gender *s* living in a specific cell *i* (in the macro area $y_i$ that includes *i*) in 2011, while $P_{y_i,t,a,s}$ is the population of a certain age class *a* and given gender *s* living in the macro area $y_i$ at time *t*. To estimate the population distribution in the years to come, we assume that the way in which the population is distributed within each macro-area does not change with time. Consequently, this formulation is also used to estimate future variations in the number of people living in each cell *i*. We recognize this as a limitation of our study.

2 The outcomes in Column 3 (Table 4) are derived from comparing the current level of accessibility with the level that would result from using projections of the age class distribution while keeping the levels of capacity and population as constant at their present-day values. In Column 3, the number of individuals of a certain age class *a* and given gender *s* living in location *i* at time *t* ($\tilde{P}_{i,t,a,s}$) is computed as follows: $\frac{P_{i,t,a,s}}{\sum _{a \in A} P_{i,t,a,s}}\cdot {\sum _{a \in A} P_{i,t_0,a,s}}$. Here, $t_0$ represents the current year (2021), and *t* corresponds to projections for +5, +10, and +15 years. Population projections, disaggregated by age class and gender ($P_{y_i,t,a,s}$), are sourced from the regional dataset [14]. The allocation of individuals to each cell *i*, necessary to derive $P_{i,t,a,s}$, followed the steps described in Footnote 1.

3 This value can also be obtained by testing the model under the assumption of an extremely high (potentially infinite) hourly availability of new GPs allocated to CHCs (i.e., $Q^{tot}$). This motivates the specific header used, which also facilitates comparison with Strategy 2, as presented in Section 4.2.1—Table 6.

4 Recall that choosing a very high value for $\alpha$ means that Constraints (M2.6) are not binding, i.e., we can potentially reallocate the availability of all the GPs . To this end, it clearly suffices to set $\alpha = 1.00$ in the case of *Strategy 2*. Nonetheless, we keep the notation “$\alpha \rightarrow + \infty$” in Table 6 to ensure consistency with the results obtained under *Strategy 1*.

5 To investigate whether focusing on vulnerable populations reveals different findings, we repeated our analysis for the elderly (i.e., people aged 65 or older), who represent more than 53% of the population in the AS-IS scenario. However, the distribution of the elderly across differently urbanized areas closely resembles that of the overall population, resulting in qualitatively and quantitatively similar findings. Therefore, for the sake of brevity, we decided not to include this analysis in the paper.
